# Heteroscedastic Calibration Using Analyzed Reference Materials as Calibration Standards

**DOI:** 10.6028/jres.093.038

**Published:** 1988-06-01

**Authors:** Robert L. Watters, Raymond J. Carroll, Clifford H. Spiegelman

**Affiliations:** National Bureau of Standards, Gaithersburg, MD 20899; Department of Statistics, Texas A&M University, College Station, TX 77843

Many instrumental analytical techniques exhibit a definable relationship between instrument response and analyte concentration over wide concentration ranges. This response is usually fit to an accepted model during the calibration phase of the measurement process. Often the calibrated concentration range (*x* values) is such that the measured response (*y* values) exhibits non-constant variance. The use of weighted regression techniques to properly estimate model parameters for this case has been described for a number of analytical applications. An inherent, if not stated, assumption in these treatments is that negligible error resides in the concentrations of the calibration standards.

A separate issue regarding calibration is the desire to minimize bias in the analysis by using calibration standards that are matched to the sample to be analyzed. It has been suggested that analyzed reference materials (ARMs) of a chemical matrix similar to that of the sample be used as calibration standards. Since the concentrations of analytes in these materials are estimates from measurements with error, using ARMs as calibration standards leads to errors in both *x* and *y* values for fitting the model. Therefore, the standard regression assumptions are not valid. A number of schemes have been developed for treating the calibration problem where both *x* and *y* have errors. However, when this problem is combined with heteroscedastic calibration, appropriate procedures are more complex.

We have recently reported an approach to heteroscedastic calibration that yields multiple-use calibration estimates and confidence intervals [1]. The first step is to obtain calibration data from Standards, which provide both estimates of the instrument response and its variability over the concentration range of interest. These estimates of uncertainty are Fitted to a model for errors in *y*,(*σ_y_*) in an iterative fashion. Each iteration is a weighted fit of the error model. The weights, $1/\sigma_{y}^{2}$, are calculated from the estimates of *σ_y_* from the previous Fit. Once the coefficients of the error model are obtained, a Final set of *σ_y_*’s is calculated and used for the weighted fit of the calibration curve. Uncertainty bands over the calibrated range are then constructed by combining the uncertainty interval for successive measurements of unknown samples and the calibration band uncertainty. Concentration estimates and confidence intervals for the unknowns can then be obtained (cf. ref. [1], fig. 3). To combine this approach with the problem of errors in *x*, we apply adjustments to both the error model fit and the calibration curve fit. The matrices used in the calculations contain standards concentration data, error estimates for both *y* and *x*, the estimated calibration curve slope, and the coefficients for *y*-error model.

Care must be exercised in using this approach, because the general problem of calibrating with analyzed reference materials can violate some key assumptions regarding the calibration model. Analyzed reference materials are often complex solids that may be impossible to completely dissolve. Reference value estimates and uncertainties cannot be used for analytes lost in the dissolution process. Furthermore, preparation of calibration standards with analyte concentrations at various levels over the range of calibration will also result in matrix concentrations that also vary. If chemical matrix-matched calibration is required to reduce systematic errors in the analysis, various dilutions of analyzed reference materials are likely to change the slope of the calibration curve at each calibrated point. When analyzed reference materials are diluted into a constant chemical matrix, this type of calibration may be appropriate. Examples of this would include the mixture of geological materials in an excess of fusion flux for dissolution or the dilution of wear metals in oil standards in a constant excess of organic solvent.
